# α-Galactosidase A Augmentation by Non-Viral Gene Therapy: Evaluation in Fabry Disease Mice

**DOI:** 10.3390/pharmaceutics13060771

**Published:** 2021-05-21

**Authors:** Julen Rodríguez-Castejón, Ana Alarcia-Lacalle, Itziar Gómez-Aguado, Mónica Vicente-Pascual, María Ángeles Solinís Aspiazu, Ana del Pozo-Rodríguez, Alicia Rodríguez-Gascón

**Affiliations:** Pharmacokinetic, Nanotechnology and Gene Therapy Group (Pharma Nano Gene), Centro de investigación Lascaray ikergunea, Faculty of Pharmacy, University of the Basque Country UPV/EHU, Paseo de la Universidad 7, 01006 Vitoria-Gasteiz, Spain; julen.rodriguez@ehu.eus (J.R.-C.); ana.alarcia@ehu.eus (A.A.-L.); itziar.gomez@ehu.eus (I.G.-A.); monica.vicente@ehu.eus (M.V.-P.); marian.solinis@ehu.eus (M.Á.S.A.); Bioaraba, 01006 Vitoria-Gasteiz, Spain

**Keywords:** gene therapy, non-viral vectors, solid lipid nanoparticles, pDNA, Fabry disease, Fabry mice, α-galactosidase A, intravenous administration

## Abstract

Fabry disease (FD) is a monogenic X-linked lysosomal storage disorder caused by a deficiency in the lysosomal enzyme α-Galactosidase A (α-Gal A). It is a good candidate to be treated with gene therapy, in which moderately low levels of enzyme activity should be sufficient for clinical efficacy. In the present work we have evaluated the efficacy of a non-viral vector based on solid lipid nanoparticles (SLN) to increase α-Gal A activity in an FD mouse model after intravenous administration. The SLN-based vector incremented α-Gal A activity to about 10%, 15%, 20% and 14% of the levels of the wild-type in liver, spleen, heart and kidney, respectively. In addition, the SLN-based vector significantly increased α-Gal A activity with respect to the naked pDNA used as a control in plasma, heart and kidney. The administration of a dose per week for three weeks was more effective than a single-dose administration. Administration of the SLN-based vector did not increase liver transaminases, indicative of a lack of toxicity. Additional studies are necessary to optimize the efficacy of the system; however, these results reinforce the potential of lipid-based nanocarriers to treat FD by gene therapy.

## 1. Introduction

Using genetic material to treat different diseases has been in the spotlight of therapeutic approaches over the last few years. In 2003 the China Food and Drug Administration (CFDA) approved the first gene therapy-based product (Gendicine™) to treat head and neck carcinoma [1]. Since then, the number of clinical trials has increased steadily, resulting in various gene therapy medical products (GTMPs) entering the market. To date, about 15 GTMPs, based on in vivo and ex vivo strategies, have been approved worldwide [2]. In addition, more than 3100 clinical trials with nucleic acids have been completed, are ongoing or have been approved [3], with a future promising outlook.

Gene therapy offers a unique opportunity especially for the treatment of monogenic diseases [4], which result from modifications in a single gene, such as Fabry disease (FD). FD is an X-linked lysosomal storage disorder caused by mutations in the *GLA* gene, leading to a deficiency in the lysosomal enzyme α-Galactosidase A (α-Gal A). As a result, glycosphingolipids, predominantly globotriaosylceramide (Gb3) and its deacylated derivative globotriaosylsphingosine (lyso-Gb3), accumulate systemically, particularly in vascular endothelial and smooth muscle cells [5]. Current available treatment options for FD include intravenous (IV) enzyme replacement therapy (ERT) with two recombinant enzymes, agalsidase α (Replagal^®^) or agalsidase β (Fabrazyme^®^). However, ERT shows important drawbacks: considerable clinic variation, the effect is determined by the initiation age, the high cost, the formation of antibodies against the infused enzyme and the biweekly IV administration frequency [6]. More recently, the approval of an oral chaperone, Migalastat^®^, has increased therapeutic options for patients with FD. This small molecule reversibly binds to the active site of specific mutant forms of endogenous α-Gal A, stabilizing it and directing it to lysosomes. Nevertheless, Migalastat^®^ is only effective in patients with certain mutant forms of α-Gal A that are amenable to the treatment [7,8].

Different strategies have been proposed as an alternative to those treatments, such as substrate reduction therapy, stem cell-based therapy, gene therapy and removal of storage material [9,10]. FD is a great candidate to be treated by gene therapy, as it is caused by the disruption of a single gene, clinical effect can be achieved with a 10% activity of normal levels, it offers long-term therapeutic effects, reducing repeated administration necessity [11], and in the case of non-viral gene therapy, it is cheaper than enzymatic therapy. At present, several clinical studies are evaluating the safety of gene therapy for FD, which differ in their therapeutic approaches [12]. The first multicenter, multinational, open-label study (NCT03454893, NCT02800070; AVR-RD-01, AVROBIO) is based on ex vivo transduction of hematopoietic stem cells with lentivirus. The aim of this approach is to transfect hematopoietic stem cell-derived cells and use them as a production platform for functional α-Gal A production, which will be secreted to the plasma and subsequently internalized by α-Gal A-deficient cells. Other two clinical studies (NCT04046224, NCT04040049) are based on adeno-associated viral vectors (AAV) for in vivo transduction of hepatocytes, using these cells as an α-Gal A-secreting platform instead of hematopoietic stem cell-derived cells; one of them (NCT04040049) is still in recruitment phase. A third clinical study on AAV-based gene therapy is also in the recruitment phase and uses an attenuated AAV (4D-310; 4D Molecular Therapeutics). Preclinical studies in mice demonstrated that the novel capsid 4D-C102 was especially efficient in transducing human cardiomyocytes.

Success of gene therapy depends largely on the delivery system used, which must ensure protection of the genetic material against degradation, facilitating its internalization and intracellular delivery into the target cells [13]. Considering the natural transduction properties of most viruses, viral vectors have been at the forefront of gene delivery systems for many years, and most of the approved GTMPs consist of viruses as gene delivery vectors [2]. Nevertheless, the oncogenic and immunogenic potential related to the delivery of viral vectors limit their use for gene therapy applications. Advances in material sciences have prompted the development of safer delivery systems. Non-viral vectors are poorly immunogenic and their production is simpler, cheaper and more reproducible. Moreover, unlike viral vectors, non-viral systems are not limited by the molecular size of the gene to be packaged. Their low transfection efficacy remains a challenge to be overcome, although it has improved by different strategies [14,15].

Lipid-based systems are the most studied non-viral vectors at clinical level [3]. Among them, lipid nanoparticles (LNs) show several advantages, such as low or absence of in vivo toxicity, good long-term stability, production by economic and solvent-free techniques and the possibility to autoclave and sterilize [16]. Even though most non-viral vectors remain at pre-clinical and clinical level, LNs have taken a step forward with the approval of Onpattro^®^ (Patisiran). This GTMP is a short interfering RNA (siRNA) formulated in LNs targeting the liver to reduce the levels of transthyretin; the Food and Drug Administration (FDA) and the European Medicines Agency (EMA) approved it in 2018 for the treatment of hereditary transthyretin amyloidosis [17]. More recently, some of the accepted vaccines to defeat the SARS-CoV-2 contain messenger RNA (mRNA) encapsulated in LNs [18]. LNs have also been used as the delivery system of mRNA encoding α-Gal A, to target hepatocytes. Once expressed, the α-Gal A was released to systemic circulation, and it was able to reduce Gb3 and lyso-Gb3 levels in heart and kidney of mice and non-human primates after repeated administration of the LNs (Moderna Inc and Translate Bio) [19,20]. Our research group has previously demonstrated the capacity of Solid Lipid Nanoparticles (SLNs) to transfect a plasmid DNA (pDNA) encoding a reporter protein (Green Fluorescent Protein, GFP) in the liver after IV administration to mice [21,22].

The objective of the present work was to evaluate the ability of a SLN-based non-viral vector carrying a pDNA encoding α-Gal A to increase in vivo enzyme activity levels in plasma and tissues after IV administration to an α-Gal A knockout (KO) mouse model of FD. Additionally, Aspartate Aminotransferase (AST) and Alanine Aminotransferase (ALT) activity were measured in the liver of mice to assess the potential toxicity of the SLN-based vector.

## 2. Materials and Methods

### 2.1. Materials

Precirol^®^ ATO 5 (glyceryl palmitostearate) was kindly provided by Gattefossé (Madrid, Spain). 1,2-Dioleoyl-3-trimethylammonium-propane chloride salt (DOTAP) was purchased from Avanti Polar-lipids, Inc. (Alabaster, AL, USA). Tween 80 and dichloromethane were obtained from Panreac (Madrid, Spain). d-(+)-Trehalose dihydrate, Protamine sulfate salt from salmon (Grade X) (P), dextran (Mn of 3380 Da) (DX), 4-methylumbelliferyl-α-d-galactopyranoside (4-MU-α-Gal), N-acetyl-d-galactosamine, 4-methylumbelliferone (4-MU) and Aspartate Aminotransferase (AST) and Alanine Aminotransferase (ALT) Activity Assay Kits were obtained from Sigma-Aldrich (Madrid, Spain). Micro BCA™ Protein Assay Kit was acquired from Thermo Fisher Scientific (Madrid, Spain). Plasmid pR-M10-αGal A was purchased from Origene (Rockville, MD, USA). Other chemicals, if not specified, were reagent grade from Sigma-Aldrich (Madrid, Spain) and Panreac (Barcelona, Spain). Fabry mice (B6;129-Glatm1Kul/JAX stock #003535) [23] were purchased from The Jackson Laboratory (Bar Harbor, ME, USA).

### 2.2. Preparation of SLNs and Vector

The SLNs were produced by a solvent emulsification–evaporation technique, previously described by del Pozo-Rodríguez et al. [24], with minor modifications. Briefly, Precirol^®^ ATO 5 was dissolved in dichloromethane (5% *w*/*v*), and then emulsified in an aqueous phase containing DOTAP (0.4% *w*/*v*) and Tween 80 (0.1% *w*/*v*). The emulsion was obtained by sonication (Branson Sonifier 250, Danbury, Connecticut, USA) for 30 s at 50 W. The organic solvent was evaporated by stirring for 1 h and 45 min of vacuum. After the evaporation of the organic solvent a suspension of SLNs was formed upon solidification of the Precirol^®^ ATO 5 in the aqueous medium. The SLNs were washed by centrifugation (3000 rpm, 20 min, 3× *g*) using Millipore (Madrid, Spain) Amicon^®^ Ultra centrifugal filters (NMWL: 100,000 Da). Finally, SLNs were lyophilized at −50 °C and 0.2 mbar for 42 h (LyoBeta 15, Telstar, Spain) in the presence of 5% (*w*/*v*) d-(+)-Trehalose dihydrate as cryoprotectant.

To obtain the vector, first, an aqueous solution of protamine (P) was added to the plasmid DNA (pDNA) pR-M10-αGal A to form P-pDNA complexes at *w*/*w* ratio of 2:1. Then, an aqueous solution of dextran (DX) was incorporated at DX-P-pDNA ratios of 1:2:1 (*w*/*w*/*w*). Finally, lyophilized SLNs were resuspended with the suspension of DX-P-pDNA complexes and incubated for 20 min at *w*/*w*/*w*/*w* ratio of 1:2:1:5. Electrostatic interactions between negative and positive charges of the components led to the formation of the final DX-P-pDNA-SLN vector, in which DX-P-pDNA complexes were adsorbed on the surface of the SLNs. The final composition of the SLN-based vector is based on previous studies [21,25,26,27,28], in which different vectors carrying pDNA encoding GFP or α-Gal A were characterized in terms of encapsulation efficacy, capacity to bind, protect and release the gene cargo, Transmission Electron Microscopy (TEM), stability, in vitro transfection efficacy, cellular uptake and cell viability.

### 2.3. Characterization of the Vector: Size, Polydispersity Index and ζ Potential Measurements

The size and polydispersity index were determined by Dynamic Light Scattering (DLS). ζ potential was measured by Laser Doppler Velocimetry (LDV). Samples were diluted in Milli-Q™ water (EDM Millipore, Billerica, MA, USA) and measurements were carried out in a ZetaSizer Nano ZS (Malvern Instruments, Worcestershire, UK).

### 2.4. Animal Model

α-Gal A KO mice (B6;129-Glatm1Kul/JAX stock #003535) were used as FD model animals. α-Gal A KO mice were generated by replacing exon III and intron III of the *GLA* gene with a neo cassette [23]. Breeding pairs were mated following the mating recommendations from The Jackson Laboratory and their offspring were genotyped by the investigation general services (SGIker) from the University of the Basque Country UPV/EHU following the genotyping protocol from The Jackson Laboratory website. Animals were housed under controlled temperature, humidity and 12 h light/dark cycles, with ad libitum access to standard rodent chow and water.

### 2.5. Animal Experimentation

In vivo studies were approved by the Ethics Committee on Animal Experimentation (CEEA) of the University of the Basque Country UPV/EHU (Permit number: M20/2017/157; approval date: 14/06/2018) following the Spanish and European Union (EU) laws and all the procedures were followed in accordance.

Procedures were conducted with ≈8-week-old male mice weighing between 20 and 28 g. Animals were divided into 8 different experimental groups (3–5 animals per group):

- Wild-type (+/0 for α-Gal A);
- Non-treated Fabry mice (−/0 for α-Gal A);
- Fabry mice treated with a single administration of the naked pDNA and sacrificed at day 3;
- Fabry mice treated with a single administration of the naked pDNA and sacrificed at day 7;
- Fabry mice treated with the multiple-dosage regimen of the naked pDNA and sacrificed 7 days after the last administration;
- Fabry mice treated with a single administration of the SLN-based vector and sacrificed at day 3;
- Fabry mice treated with a single administration of the SLN-based vector and sacrificed at day 7;
- Fabry mice treated with the multiple-dosage regimen of the SLN-based vector and sacrificed 7 days after the last administration.

**Figure 1:**
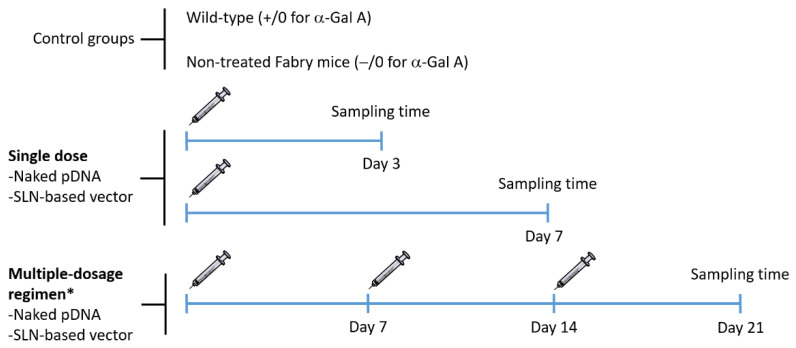
Schematic representation of the experimental design of the administration and sampling protocol. * One dose per week for 3 weeks. α-Gal A: α-Galactosidase A; pDNA: plasmid DNA; SLN: solid lipid nanoparticle.

Figure 1 features the experimental design of the administration and sampling protocol. In order to avoid distress during experimental manipulation, mice were anesthetized with 1–2% isoflurane (IsoFlo, Abbott, Madrid, Spain) in air, at a flow rate of 0.5–1 L/min. Following the standard procedure, a unique dose of the naked pDNA and the SLN-based vector was injected into the tail vein in a volume of 100 µL (60 µg of plasmid) for single-dose studies. For multiple-dose studies, the same dose was injected once a week, for 3 weeks. The selection of the dose of pDNA was based on previous in vivo studies, where protein expression was detected after the administration of SLNs containing 60 µg of pDNA encoding GFP [21,22,29].

Three and seven days after the single administration, and 7 days after the last administration for those receiving the multiple-dosage regimen, mice were humanely euthanatized by cervical dislocation. Blood was collected by cardiac puncture on euthanized animals, centrifuged at 5000 rpm at 4 °C for 8 min to obtain plasma, which was stored at −80 °C. Heart, kidney, liver, spleen and brain were harvested from each mouse and stored at −80 °C for analysis. Samples collected from wild-type mice and non-treated Fabry mice were used as control.

### 2.6. α-Galactosidase A Activity Assay

Enzymatic activity of α-Gal A was determined by a fluorimetric assay based on the conversion of 4-methylumbelliferyl-α-d-galactopyranoside (4-MU-α-Gal) into the product 4-methylumbelliferone (4-MU). Tissues were homogenized by an MT-3K mini handheld homogenizer (Hangzhou Miu Instruments Co., Ltd., Hangzhou, China) and centrifuged at 12,000× *g* at 4 °C for 10 min. Supernatants were collected for the assay. Plasma samples were used directly. An aliquot of each sample was incubated with 4-MU-α-Gal (5 mM) and N-acetyl-d-galactosamine (100 mM), a specific inhibitor of α-N-acetylgalactosaminidase (α-Galactosidase B), in 0.1 M sodium citrate buffer (pH = 4.4) at 37 °C under agitation for pre-determined periods of time, depending on the sample. The reaction was stopped with 0.1 M glycine-NaOH buffer (pH = 10.4). The resultant 4-MU was determined by measurement of fluorescence (λexcitation = 360 nm; λemission = 450 nm). The protein concentrations were determined by the Micro BCA™ protein assay. One unit of α-Gal A activity is equivalent to the hydrolysis of 1 nmol of the substrate 4-MU-α-Gal in 1 h at 37 °C. α-Gal A activity was expressed as 4-MU nmol/h/mg total protein or 4-MU nmol/h/mL plasma.

### 2.7. Liver Transaminase Activity Assay

To assess the immunogenicity of the naked pDNA and SLN-based vector, the activity of Aspartate Aminotransferase (AST) and Alanine Aminotransferase (ALT) were measured in the liver homogenates of the mice by a colorimetric test, in accordance with the manufacturer’s instructions (Sigma-Aldrich, Madrid, Spain).

### 2.8. Data Analysis and Statistics

Normal distribution of data was assessed by Shapiro–Wilk test and homogeneity of variance by the Levene test. Student’s t-test was used to compare means from two independent groups and ANOVA for multiple comparisons followed by Bonferroni or T3 Dunnett post hoc, depending on the results of the Levene test of homogeneity of variances. *p* < 0.05 was considered statistically significant. All statistical computations were performed with IBM SPSS Statistics 26 (IBM Corp, Armonk, NY, USA). Data are shown as mean ± standard deviation (SD).

## 3. Results

### 3.1. Characterization of the Vector: Size, Polydispersity Index and ζ Potential Measurements

The particle size of the vector was 233 ± 10.5 nm and it showed a polydispersity index of 0.4 ± 0.02. The surface presented a positive charge of +43 ± 1.0 mV.

### 3.2. α-Galactosidase A Activity Assay

**Figure 2:**
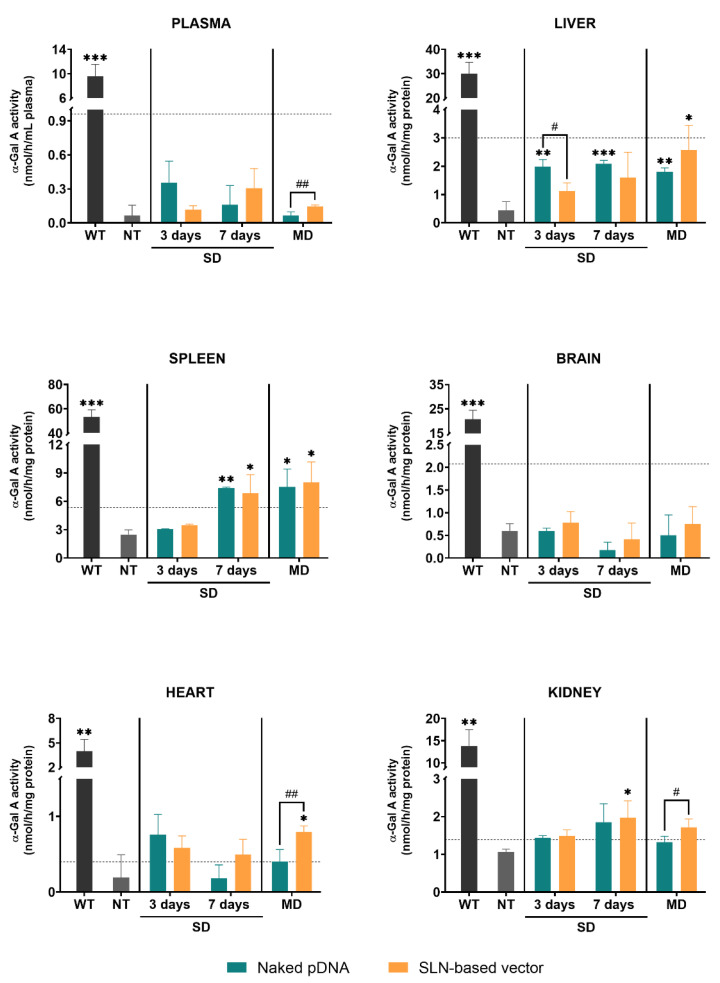
α-Galactosidase A activity in plasma and tissues of wild-type, non-treated and treated Fabry mice with the naked pDNA or the SLN-based vector, 3 and 7 days after a single dose and 7 days after the last administration of the multiple-dosage regimen. Data are expressed as mean ± SD (*n* = 3–5 per group). * *p* < 0.05, ** *p* < 0.01, *** *p* < 0.001 with respect to the non-treated group; # *p* < 0.05, ## *p* < 0.01 between the naked pDNA and the SLN-based vector. Horizontal discontinuous lines refer to 10% of the α-Galactosidase A activity of the wild-type. α-Gal A: α-Galactosidase A; WT: wild-type; NT: non-treated; SD: single dose; MD: multiple doses; pDNA: plasmid DNA; SLN: solid lipid nanoparticle.

Figure 2 represents the α-Gal A activity in different tissues of wild-type, non-treated and treated Fabry mice with either the naked pDNA or the SLN-based vector. After a single administration, the enzyme activity was measured at day 3 and 7. When animals received three doses, the enzyme activity was measured 7 days after the last dose.

Untreated Fabry mice showed significantly lower α-Gal A activity levels in plasma and all tissues as compared to the wild-type. α-Gal A activity in plasma of Fabry mice was significantly higher in the animals treated with three doses of the SLN-based vector in comparison to the animals treated with the naked pDNA. In liver, α-Gal A activity of mice treated with the naked pDNA was significantly higher compared to non-treated Fabry mice. Only when mice were treated with three doses of the SLN-based vector α-Gal A activity was significantly higher than in non-treated animals. In spleen, 7 days after the single administration and the multiple-dosage regimen of both the naked pDNA and the SLN-based vector, enzyme activity significantly increased with respect to non-treated mice. In heart, a significantly higher α-Gal A activity was only found when animals were treated with three doses of the SLN-based vector. This increment also resulted in a significant difference with respect to the administration of three doses of the naked pDNA. In kidney, 7 days after the administration of the SLN-based vector to FD mice, α-Gal A activity was higher than in the non-treated group. Three doses of the vector produced a greater increment of the enzyme activity compared to the naked pDNA. In heart and kidney, the administration of the naked pDNA did not significantly increase the enzyme activity. Neither the administration of the naked pDNA nor the administration of the SLN-based vector induced any change of the α-Gal A activity in the brain of the animals.

### 3.3. Liver Transaminase Activity Assay

**Figure 3:**
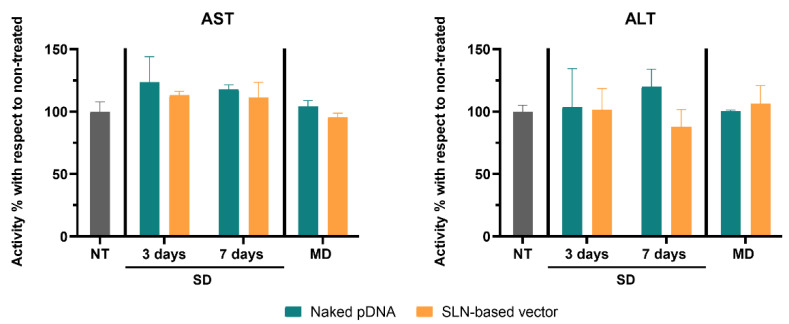
Aspartate Aminotransferase (AST) and Alanine Aminotransferase (ALT) activity percentage increment in livers of treated Fabry mice with respect to untreated mice, 3 and 7 days after a single administration of the naked pDNA and the SLN-based vector, and 7 days after the last administration of the multiple-dosage regimen. Data are expressed as mean ± SD (*n* = 3–5 per group). NT: non-treated; SD: single dose; MD: multiple doses; pDNA: plasmid DNA; SLN: solid lipid nanoparticle.

Figure 3 shows AST and ALT activity levels in the livers of mice. The administration of the naked pDNA or the SLN-based vector did not significantly increase AST and ALT activity, neither with a single injection nor with multiple doses.

## 4. Discussion

Endogenous production of α-Gal A by gene therapy allows a hopeful perspective for the treatment of FD. Even though recent studies with mRNA administered to animal models have shown promising results [19,20], pDNA may provide a much more sustained and long-lasting gene expression. Other advantages of using pDNA versus mRNA include the possibility to obtain abundant purified pDNA easily and economically, and the opportunity to perform repeated administrations without triggering an immune response [30]. Nevertheless, pDNA transfection is more difficult than that of mRNA, mainly due to the nuclear entry barrier [25]. In earlier studies, our research group has demonstrated the potential of SLN-based vectors-mediated pDNA delivery encoding α-Gal A to produce the enzyme in a liver-derived cell line (Hep G2) [26] and in a cell model of FD [27]. Herein, we report the ability of the system to increase α-Gal A activity in plasma and different tissues after IV administration to a mouse model of FD.

The vector, prepared with DX, P, pDNA encoding α-Gal A (pR-M10-αGal A) and SLNs, presents suitable characteristics for in vivo administration: particle size in the nanometer range (233 ± 10.5 nm), and a positive surface charge (+43 ± 1.0 mV). In a previous work, we showed that the DX-P-pDNA-SLN vector lacked of erythrocytes agglutination effect and hemolytic activity [21]. Additionally, in the mentioned work, the vector bearing a pDNA that encodes the intracellular reporter protein GFP was able to transfect the liver, lung and spleen of mice after IV administration.

In order to know the capacity of the SLN-based vector to induce an increment in α-Gal A activity in vivo, we administered it to *GLA* gene KO mice. In addition to the genotype of Fabry mice, we also confirmed the phenotype in terms of α-Gal A activity levels as a useful model to evaluate the efficacy of formulations to increase in vivo enzymatic activity. The quantification of the α-Gal A activity in plasma and tissues of FD mice agreed with those published by other researchers [19,20,30,31], and it was significantly lower than the levels obtained in the same strain wild-type for *GLA* (Figure 2).

FD phenotypes are directly associated to the residual α-Gal A activity. In males, the classic phenotype is the most severe due to low (<3%) α-Gal A activity; however, other mutations present higher enzyme activity and cause late-onset or attenuated pathology [32]. In this sense, restoring the enzyme activity to levels of healthy individuals does not seem necessary, and clinical improvement can be obtained with a relatively small increment [33,34]; it has been demonstrated that 10% enzyme activity of the wild-type is sufficient to totally clear deposit of Gb3 in various organs [35]. Different studies have reported an efficient reduction of Gb3 and lyso-Gb3 and maintenance of the reduced amount even after the α-Gal A levels fell [19,20,30]. We assessed the enzyme activity to ensure that the produced enzyme was functional.

After IV administration of the SLN-based vector to Fabry mice the α-Gal A activity significantly increased in liver, spleen, heart and kidney with respect to non-treated mice. It is known that nanoparticles greater than 200 nm in diameter, accumulate predominantly in the liver and spleen [36]. In this sense, in a previous work carried out by our research group [37], the IV administration of radiolabeled LNs formulated with Precirol^®^ ATO 5 and Tween 80 to rats provided the highest radioactivity levels in kidney, liver and spleen.

The administration of three doses of the SLN-based vector augmented α-Gal A activity in liver to levels about 10% of that of the wild-type, maybe due to the accumulation of the vector and the subsequent sustained release of the pDNA. Moreover, administration of the SLN-based vector resulted in an increase in α-Gal A activity that reached around 14% and 20% of the wild-type in kidney and heart, respectively; it is important to consider that affectation of these organs is the major cause of morbidity and mortality in Fabry patients [38,39,40]. In those tissues, enzyme activity incremented significantly above the naked pDNA after multiple doses. The naked pDNA and the SLN-based vector produced a significant increment of α-Gal A activity in spleen 7 days after both, single administration and multiple doses. The spleen is one of the major organs responsible for filtering the blood of foreign material [41], and previous investigations have demonstrated the high uptake of nanocarriers by this tissue [42]. Nanoparticles are commonly scavenged by monocytes and macrophages, which are part of the reticuloendothelial system and often accumulate in the liver and spleen [42]. The transfection of these cells may justify the low levels in plasma despite the enzyme activity levels observed in liver and spleen.

The naked pDNA was able to increase α-Gal A activity of Fabry mice not only in spleen, but also in liver. Intravenously administered naked nucleic acids show very short circulating half-life [43]. It has been shown that the liver is the main organ responsible for the rapid clearance of systemically administered pDNA [44,45]. Therefore, the protein expression observed in liver during the 7 days after the injection of the naked pDNA is maybe due to a rapid removal from the bloodstream by this organ. Multiple administrations of the naked pDNA did not lead to a higher activity level than that observed with a single injection but remained approximately equal.

Accumulation of lipids in the liver can lead to hepatic toxicity [46] and activate an immune response. Considering the α-Gal A activity detected in the liver after the administration of the naked pDNA and the SLN-based vector, indicative of the access to this organ, we measured the activity of liver transaminases to assess the immunogenicity. An increase in liver transaminases is indicative of liver injury, and AST and ALT are the most widely used parameters [47,48]. Neither the naked pDNA nor the SLN-based vector increased the activity of liver transaminases with respect to non-treated mice.

The increase of α-Gal A activity observed after IV administration to mice of either the naked pDNA or the SLN-based vector, demonstrate their ability to circulate in the bloodstream and reach the tissues. Contrary to the naked pDNA, the SLN-based vector provides a higher stability to the pDNA and facilitates its internalization by the cells, which may explain the higher efficacy [21,25,26,27,28]. After the administration of the SLN-based vector once a week for three weeks, heart and kidney (the most important target organs) were the tissues where the greatest difference in α-Gal A activity between the naked pDNA and the SLN-based vector was detected. Furthermore, one of the major advantages of SLN-based vectors is the versatility they offer to modify their surface [49]. Taking advantage of that property, vectors could be formulated to direct the therapy by active targeting to organs with major affectation. Thereby, depending on the phenotype and the most affected organs in each patient, the treatment could be personalized and direct it to the desire site. Alternatively, selective transfection of a specific tissue that could efficiently express α-Gal A can be considered. Hydrolytic enzymes implicated in lysosomal storage disorders, including α-Gal A, are secreted and can reach neighboring cells and distant tissues via systemic circulation. Therefore, transfection of a certain organ can lead to a “cross-correction” phenomenon [33]. In this regard, the liver is a highly specialized organ in protein production that could act as α-Gal A factory to overexpress the enzyme, so that it is released to the bloodstream to reach and correct distant tissues [26]. For hepatocyte-targeted therapy, the particle size of the carrier is a key factor [50], and therefore, a smaller vector must be developed. Furthermore, non-viral vectors can be directed to hepatocytes by incorporating sugar ligands whose receptors are abundantly expressed in the surface of hepatocytes, such as the asialoglycoprotein receptor, which effectively binds and internalizes oligosaccharides and glycoproteins presenting D-galactose and N-acetyl-D-galactosamine moieties [51].

Brain affectation in FD involves manifestations that can vary in degree and extent, and are mainly characterized by the development of white matter hyperintensities, stroke and ischemia attacks [52]. Although nanocarriers have been proposed for targeting bioactives to the brain [40], overcoming the blood–brain barrier (BBB) is still challenging. In our study, we did not detect an increase of α-Gal A activity in brain, neither with the naked pDNA nor with the SLN-based vector. The inability of the vector to cross the BBB may probably explain the lack of effect in the brain. In addition, although circulating enzyme in blood could reach other tissues, none of the existing recombinant enzymes available for the treatment of FD has demonstrated the ability to reach this organ. Functionalization of nanocarriers is helpful, if not necessary, to deliver bioactives to the brain. Insulin and transferrin are usually employed as brain targeting moieties, because these proteins present abundant receptors in BBB endothelial cells. Muntoni et al. [53] have recently developed Methotrexate-loaded SLNs functionalized with insulin and transferrin by a PEGylated-maleimide linker, and SLNs were successfully targeted towards the BBB in vivo in rats. Hence, in order to promote the effect of our vector in the brain, decoration with the proteins insulin or transferrin could be considered in future works.

Although reduction of Gb3 and lyso-Gb3 deposits should be confirmed, the SLN-based vector was able to partially restore α-Gal A activity in some tissues. In a previous study [20], the administration of the ERT recombinant α-Gal A to Fabry mice led to a very rapid decrease in serum (no enzyme was observed in the sera 6 h after the IV injection). In other studies of Fabry mice, IV administration of mRNA encapsulated in LNs increased α-Gal A activity to supraphysiological levels. In one of these studies [20], the expression of the enzyme decreased as soon as the mRNA was degraded, giving rise to the need for repeated administrations to maintain reduced substrate levels. In another study [19], α-Gal A activity was maintained up to 28 days after a single dose. pDNA-based non-viral gene therapy has not revealed relevant results so far [30,54], despite it presents several advantages with respect to mRNA. In this regard, apart from the higher stability, the persistence of pDNA in the nucleus as an episome may provide a long-lasting enzyme expression, although optimization of the vector results essential for this purpose.

## 5. Conclusions

The results show the ability of SLN-based non-viral vectors to increase α-Gal A activity in different organs, especially in liver, spleen, kidney and heart, after IV administration to FD mice. The administration of a dose per week for three weeks was more effective than a single-dose administration. Importantly, no liver toxicity was induced, allowing the perspective of a liver-directed gene therapy. Although transfection efficacy of our vector should be improved, this study is a proof of concept that shows its potential to treat FD by gene therapy.

## References

[1] Zhang W.W., Li L., Li D., Liu J., Li X., Li W., Xu X., Zhang M.J., Chandler L.A., Lin H. (2018). The First Approved Gene Therapy Product for Cancer Ad-p53 (Gendicine): 12 Years in the Clinic. Hum. Gene Ther..

[2] Wang F., Qin Z., Lu H., He S., Luo J., Jin C., Song X. (2019). Clinical translation of gene medicine. J. Gene Med..

[3] (2021). Gene Therapy Clinical Trials Worldwide, Provided by the Journal of Gene Medicine, John Wileys and Sons LTD. https://a873679.fmphost.com/fmi/webd/GTCT.

[4] Buck J., Grossen P., Cullis P.R., Huwyler J., Witzigmann D. (2019). Lipid-based DNA therapeutics: Hallmarks of non-viral gene delivery. ACS Nano.

[5] Bernardes T.P., Foresto R.D., Kirsztajn G.M. (2020). Fabry disease: Genetics, pathology, and treatment. Rev. Assoc. Med. Bras..

[6] Miller J.J., Kanack A.J., Dahms N.M. (2020). Progress in the understanding and treatment of Fabry disease. Biochim. Biophys. Acta Gen. Subj..

[7] McCafferty E.H., Scott L.J. (2019). Migalastat: A Review in Fabry Disease. Drugs.

[8] European Medicines Agency Science Medicines Health. https://www.ema.europa.eu/en/medicines/human/EPAR/galafold.

[9] van der Veen S.J., Hollak C.E.M., van Kuilenburg A.B.P., Langeveld M. (2020). Developments in the treatment of Fabry disease. J. Inherit. Metab. Dis..

[10] Felis A., Whitlow M., Kraus A., Warnock D.G., Wallace E. (2020). Current and Investigational Therapeutics for Fabry Disease. Kidney Int. Reports.

[11] Sands M.S., Davidson B.L. (2006). Gene therapy for lysosomal storage diseases. Mol. Ther..

[12] Lenders M., Brand E. (2021). Fabry Disease: The Current Treatment Landscape. Drugs.

[13] del Pozo-Rodríguez A., Rodríguez-Gascón A., Rodríguez-Castejón J., Vicente-Pascual M., Gómez-Aguado I., Battaglia L.S., Solinís Aspiazu M.Á., Silva A.C., Moreira J.N., Lobo J.M.S., Almeida H. (2020). Gene Therapy. Advances in Biochemical Engineering/Biotechnology.

[14] Carvalho M., Sepodes B., Martins A.P. (2017). Regulatory and Scientific Advancements in Gene Therapy: State-of-the-Art of Clinical Applications and of the Supporting European Regulatory Framework. Front. Med..

[15] Rodríguez-Gascón A., del Pozo-Rodríguez A., Isla A., Solinís Aspiazu M.Á. (2015). Vaginal gene therapy. Adv. Drug Deliv. Rev..

[16] del Pozo-Rodríguez A., Solinís Aspiazu M.Á., Rodríguez-Gascón A. (2016). Applications of lipid nanoparticles in gene therapy. Eur. J. Pharm. Biopharm..

[17] Kristen A.V., Ajroud-Driss S., Conceição I., Gorevic P., Kyriakides T., Obici L. (2019). Patisiran, an RNAi therapeutic for the treatment of hereditary transthyretin-mediated amyloidosis. Neurodegener. Dis. Manag..

[18] Wu Z., Li T. (2021). Nanoparticle-Mediated Cytoplasmic Delivery of Messenger RNA Vaccines: Challenges and Future Perspectives. Pharm. Res..

[19] Zhu X., Yin L., Theisen M., Zhuo J., Siddiqui S., Levy B., Presnyak V., Frassetto A., Milton J., Salerno T. (2019). Systemic mRNA Therapy for the Treatment of Fabry Disease: Preclinical Studies in Wild-Type Mice, Fabry Mouse Model, and Wild-Type Non-human Primates. Am. J. Hum. Genet..

[20] DeRosa F., Smith L., Shen Y., Huang Y., Pan J., Xie H., Yahalom B., Heartlein M.W. (2019). Improved Efficacy in a Fabry Disease Model Using a Systemic mRNA Liver Depot System as Compared to Enzyme Replacement Therapy. Mol. Ther..

[21] Delgado D., Rodríguez-Gascón A., del Pozo-Rodríguez A., Echevarría E., Ruiz de Garibay A.P., Rodríguez J.M., Solinís Aspiazu M.Á. (2012). Dextran-protamine-solid lipid nanoparticles as a non-viral vector for gene therapy: In vitro characterization and in vivo transfection after intravenous administration to mice. Int. J. Pharm..

[22] Delgado D., del Pozo-Rodríguez A., Solinís Aspiazu M.Á., Bartkowiak A., Rodríguez-Gascón A. (2013). New gene delivery system based on oligochitosan and solid lipid nanoparticles: “In vitro” and “in vivo” evaluation. Eur. J. Pharm. Sci..

[23] Ohshima T., Murray G.J., Swaim W.D., Longenecker G., Quirk J.M., Cardarelli C.O., Sugimoto Y., Pastan I., Gottesman M.M., Brady R.O. (1997). α-Galactosidase A deficient mice: A model of fabry disease. Proc. Natl. Acad. Sci. USA.

[24] del Pozo-Rodríguez A., Delgado D., Solinís Aspiazu M.Á., Rodríguez-Gascón A., Pedraz J.L. (2007). Solid lipid nanoparticles: Formulation factors affecting cell transfection capacity. Int. J. Pharm..

[25] Gómez-Aguado I., Rodríguez-Castejón J., Vicente-Pascual M., Rodríguez-Gascón A., del Pozo-Rodríguez A., Solinís Aspiazu M.Á. (2020). Nucleic Acid Delivery by Solid Lipid Nanoparticles Containing Switchable Lipids: Plasmid DNA vs. Messenger RNA. Molecules.

[26] Ruiz de Garibay A.P., Delgado D., del Pozo-Rodríguez A., Solinís Aspiazu M.Á., Rodríguez-Gascón A. (2012). Multicomponent nanoparticles as nonviral vectors for the treatment of fabry disease by gene therapy. Drug Des. Devel. Ther..

[27] Ruiz de Garibay A.P., Solinís Aspiazu M.Á., del Pozo-Rodríguez A., Apaolaza P.S., Shen J.S., Rodríguez-Gascón A. (2015). Solid lipid nanoparticles as non-viral vectors for gene transfection in a cell model of fabry disease. J. Biomed. Nanotechnol..

[28] Apaolaza P.S., del Pozo-Rodríguez A., Torrecilla J., Rodríguez-Gascón A., Rodríguez J.M., Friedrich U., Weber B.H.F., Solinís Aspiazu M.Á. (2015). Solid lipid nanoparticle-based vectors intended for the treatment of X-linked juvenile retinoschisis by gene therapy: In vivo approaches in Rs1h-deficient mouse model. J. Control. Release.

[29] del Pozo-Rodríguez A., Delgado D., Solinís Aspiazu M.Á., Pedraz J.L., Echevarría E., Rodríguez J.M., Rodríguez-Gascón A. (2010). Solid lipid nanoparticles as potential tools for gene therapy: In vivo protein expression after intravenous administration. Int. J. Pharm..

[30] Nakamura G., Maruyama H., Ishii S., Shimotori M., Kameda S., Kono T., Miyazaki J.I., Kulkarni A.B., Gejyo F. (2008). Naked plasmid DNA-based α-galactosidase a gene transfer partially reduces systemic accumulation of globotriaosylceramide in Fabry mice. Mol. Biotechnol..

[31] Ishii S., Yoshioka H., Mannen K., Kulkarni A.B., Fan J.Q. (2004). Transgenic mouse expressing human mutant α-galactosidase A in an endogenous enzyme deficient background: A biochemical animal model for studying active-site specific chaperone therapy for Fabry disease. Biochim. Biophys. Acta Mol. Basis Dis..

[32] Nowak A., Huynh-Do U., Krayenbuehl P.A., Beuschlein F., Schiffmann R., Barbey F. (2020). Fabry disease genotype, phenotype, and migalastat amenability: Insights from a national cohort. J. Inherit. Metab. Dis..

[33] Rastall D.P.W., Amalfitano A. (2015). Recent advances in gene therapy for lysosomal storage disorders. Appl. Clin. Genet..

[34] Fan J.-Q., Ishii S. (2007). Active-site-specific chaperone therapy for Fabry disease. FEBS J..

[35] Takahashi H., Hirai Y., Migita M., Seino Y., Fukuda Y., Sakuraba H., Kase R., Kobayashi T., Hashimoto Y., Shimada T. (2002). Long-term systemic therapy of Fabry disease in a knockout mouse by adeno-associated virus-mediated muscle-directed gene transfer. Proc. Natl. Acad. Sci. USA.

[36] Moghimi S.M., Hunter A.C., Andresen T.L. (2012). Factors Controlling Nanoparticle Pharmacokinetics: An Integrated Analysis and Perspective. Annu. Rev. Pharmacol. Toxicol..

[37] Beloqui A., Solinís Aspiazu M.Á., Delgado A., Évora C., del Pozo-Rodríguez A., Rodríguez-Gascón A. (2013). Biodistribution of Nanostructured Lipid Carriers (NLCs) after intravenous administration to rats: Influence of technological factors. Eur. J. Pharm. Biopharm..

[38] Alroy J., Sabnis S., Kopp J.B. (2002). Renal Pathology in Fabry Disease. J. Am. Soc. Nephrol..

[39] Branton M.H., Schiffmann R., Sabnis S.G., Murray G.J., Quirk J.M., Altarescu G., Goldfarb L., Brady R.O., Balow J.E., Austin H.A. (2002). Natural history of fabry renal disease: Influence of α-galactosidase a activity and genetic mutations on clinical course. Medicine (Baltimore).

[40] Azevedo O., Gago M.F., Miltenberger-Miltenyi G., Sousa N., Cunha D. (2021). Review fabry disease therapy: State-of-the-art and current challenges. Int. J. Mol. Sci..

[41] Cesta M.F. (2006). Normal Structure, Function, and Histology of the Spleen. Toxicol. Pathol..

[42] Chrastina A., Massey K.A., Schnitzer J.E. (2011). Overcoming in vivo barriers to targeted nanodelivery. Wiley Interdiscip. Rev. Nanomed. Nanobiotechnology.

[43] Ruponen M., Honkakoski P., Rönkkö S., Pelkonen J., Tammi M., Urtti A. (2003). Extracellular and intracellular barriers in non-viral gene delivery. J. Control. Release.

[44] Kawabata K., Takakura Y., Hashida M. (1995). The Fate of Plasmid DNA After Intravenous Injection in Mice: Involvement of Scavenger Receptors in Its Hepatic Uptake. Pharm. Res. An Off. J. Am. Assoc. Pharm. Sci..

[45] Hisazumi J., Kobayashi N., Nishikawa M., Takakura Y. (2004). Significant role of liver sinusoidal endothelial cells in hepatic uptake and degradation of naked plasmid DNA after intravenous injection. Pharm. Res..

[46] Van Hoecke L., Roose K. (2019). How mRNA therapeutics are entering the monoclonal antibody field. J. Transl. Med..

[47] Oh R.C., Hustead T.R., Ali S.M., Pantsari M.W. (2017). Mildly Elevated Liver Transaminase Levels: Causes and Evaluation. Am. Fam. Physician.

[48] Reagan W.J., Yang R.-Z., Park S., Goldstein R., Brees D., Gong D.-W. (2012). Metabolic Adaptive ALT Isoenzyme Response in Livers of C57/BL6 Mice Treated with Dexamethasone. Toxicol. Pathol..

[49] Harms M., Müller-Goymann C.C. (2011). Solid lipid nanoparticles for drug delivery. J. Drug Deliv. Sci. Technol..

[50] Jacobs F., Gordts S.C., Muthuramu I., De Geest B. (2012). The liver as a target organ for gene therapy: State of the art, challenges, and future perspectives. Pharmaceuticals.

[51] Chen F., Huang G., Huang H. (2019). Sugar ligand-mediated drug delivery. Future Med. Chem..

[52] Cocozza S., Russo C., Pontillo G., Pisani A., Brunetti A. (2018). Neuroimaging in Fabry disease: Current knowledge and future directions. Insights Imaging.

[53] Muntoni E., Martina K., Marini E., Giorgis M., Lazzarato L., Salaroglio I., Riganti C., Lanotte M., Battaglia L. (2019). Methotrexate-Loaded Solid Lipid Nanoparticles: Protein Functionalization to Improve Brain Biodistribution. Pharmaceutics.

[54] Przybylska M., Wu I.-H., Zhao H., Ziegler R.J., Tousignant J.D., Desnick R.J., Scheule R.K., Cheng S.H., Yew N.S. (2004). Partial correction of the α-galactosidase A deficiency and reduction of glycolipid storage in Fabry mice using synthetic vectors. J. Gene Med..

